# The Comparison of General Characteristics and Early and Late Post-intervention Results in Patients With Trigeminal Neuralgia Secondary to Multiple Sclerosis and Idiopathic Trigeminal Neuralgia Treated With Radiofrequency Thermocoagulation

**DOI:** 10.7759/cureus.44810

**Published:** 2023-09-06

**Authors:** Hasan Burak Gündüz, Abdullah Safa Kurşun, Fatih Ekşi, Fikret Öztürk, Seda Yağmur Karataş Okumuş, Mesude Tütüncü, Aysun Soysal, Erhan Emel

**Affiliations:** 1 Neurological Surgery, Bakirkoy Prof. Dr. Mazhar Osman Training and Research Hospital for Psychiatric Neurological Diseases, İstanbul, TUR; 2 Neurological Surgery, Bakirkoy Prof. Dr. Mazhar Osman Training and Research Hospital for Neurology, Neurosurgery and Psychiatry, İstanbul, TUR; 3 Neurology, Bakirkoy Prof. Dr. Mazhar Osman Training and Research Hospital for Psychiatric Neurological Diseases, İstanbul, TUR

**Keywords:** radiofrequency thermocoagulation, trigeminal neuralgia secondary to multiple sclerosis, idiopathic trigeminal neuralgia, multiple sclerosis, trigeminal neuralgia

## Abstract

Aim

The aim of this study was to investigate possible differences (in terms of demographic structure, disease history, complaints, clinical findings, early and late treatment outcomes, and complications) between patients with idiopathic trigeminal neuralgia (ITN) and trigeminal neuralgia (TN) secondary to multiple sclerosis (MS) who were admitted to our clinic and underwent radiofrequency (RF) thermocoagulation procedure.

Materials and methods

Patients who underwent percutaneous radiofrequency thermocoagulation with a diagnosis of trigeminal neuralgia by a single neurosurgeon in a single neurosurgery clinic between January 2005 and January 2020 were included in this study. Patients were divided into two groups: idiopathic trigeminal neuralgia and trigeminal neuralgia secondary to multiple sclerosis (MSTN) according to their diagnosis. In our study, 215 TN patients who underwent 286 procedures were included. These patients were categorized according to age, sex, involved side, pain localization, and pain history. Postoperative complications were determined after each intervention. The early and late results of all interventions were evaluated, and the results were compared between both groups. All results were statistically analyzed.

Results

Considering the age of the patients, the mean age of the idiopathic group was higher than the multiple sclerosis group (58.18>49.46). In terms of the side of pain, bilateral involvement was significantly more common in the MS secondary group (1.48%<30.77%). There was no significant difference between the early results of both groups. In terms of remission periods, the pain-free period in the MS secondary group was significantly shorter than in the idiopathic group (mean value in months, 30.87>23.81).

Conclusion

The radiofrequency thermocoagulation of the trigeminal nerve is a highly effective, low-complication, reproducible procedure for trigeminal neuralgia, but the search for ways to improve the efficacy of treatment in MS patients should continue.

## Introduction

According to the third edition of the International Classification of Headache Disorders, trigeminal neuralgia (TN) is a disorder characterized by recurrent unilateral brief electric shock-like pains, abrupt in onset and termination, limited to the distribution of one or more divisions of the trigeminal nerve, and triggered by innocuous stimuli. It may develop without apparent cause or be a result of another diagnosed disorder [1,2].

Cruccu et al. have classified clinically established TN into three etiological categories: idiopathic TN (ITN), TN occurring without an identified cause; classical TN, TN caused by vascular compression on the trigeminal nerve root; and secondary TN, TN caused by a cerebellopontine angle tumor or a major neurological disease such as multiple sclerosis (MS) [3]. The incidence rate of idiopathic TN in the general population is approximately 12 per 100,000 persons [4,5].

MS is a chronic inflammatory disease that causes demyelination and neurodegeneration in the central nervous system [6]. Demyelinating lesions in the brainstem can result in the involvement of cranial nerves, leading to many symptoms. One of these is TN. TN is 20 times more common in patients with MS compared to the general population [7-9]. Houshi et al. [10] calculated the prevalence of TN as 3.4% in their review of 30,348 MS patients including 19 studies. The rate of MS among patients with TN was found to be between 2% and 14% [7,10,11].

The aim of this study was to investigate possible differences (in terms of demographic structure, disease history, complaints, clinical findings, early and late treatment outcomes, and complications) between patients with idiopathic trigeminal neuralgia (ITN) and trigeminal neuralgia secondary to multiple sclerosis (MSTN) who were admitted to our clinic and underwent radiofrequency thermocoagulation procedure.

## Materials and methods

Patients who underwent percutaneous radiofrequency (RF) thermocoagulation with the diagnosis of TN by a neurosurgeon in a single neurosurgery clinic between January 2005 and January 2020 were included in this study. Patients were categorized into two groups, idiopathic trigeminal neuralgia (ITN) and trigeminal neuralgia secondary to multiple sclerosis (MSTN), according to their diagnosis. Our hospital database was used for patient data. During this process, utmost care was taken not to reveal personal patient information.

All patients with ITN and MSTN were diagnosed by a neurologist and primarily treated with medical therapy. Patients who did not respond to medical treatment and developed resistance or side effects were referred to the neurosurgery clinic.

A follow-up period of 36 months was determined for each procedure. Since some patients were exposed to more than one procedure, remission times were recorded on a procedure basis rather than on a patient basis. Although the remission periods of many patients exceeded 36 months, these periods were not specified in the study. The aim of this is to standardize the follow-up periods and make the comparison results more reliable. These follow-ups were performed by outpatient clinic visits and telephone surveys.

Patients with a secondary etiology other than MS (such as intracranial tumor), patients who could not be followed up at 36 months, and patients with atypical facial pain were excluded from this study. In addition, all patients with TN underwent cranial magnetic resonance imaging examinations including appropriate sequences that could show vascular compression. Microvascular decompression surgery was recommended especially when there was evidence of vascular compression. Patients who underwent microvascular decompression surgery were excluded from the study.

Our study included 215 TN patients who underwent 286 interventions. Of these 215 patients, 202 were accepted as ITN and 13 as MSTN. These patients were categorized in terms of age, sex, involved side, localization of pain, and history of pain. These results were statistically analyzed and compared. The time between the diagnosis of MS and the onset of TN complaints has been documented in patients with MS. Early post-intervention outcomes were scored with the Barrow Neurological Institute (BNI) pain severity scale (Table 1) [12]. Postoperative complications were determined after each intervention. Within a 36-month period, the duration of pain remission and the number of re-interventions were determined. All these results were compared in two groups, and it was investigated whether the differences between the groups were statistically significant.

**Table 1:** Barrow Neurological Institute pain intensity scale

Pain score	Definition
I	No trigeminal pain, no medication
II	Occasional pain, not requiring medication
III	Some pain, adequately controlled with medication
IV	Some pain, not adequately controlled with medication
V	Severe pain, no pain relief

Statistical assessment

The obtained results were analyzed in the PC-based Statistical Package for Social Sciences (SPSS) statistical program (version 16.0) (SPSS Inc., Chicago, IL), and the differences between the two groups were reviewed. Primarily, the normality analysis of data distributions was performed, and skewness and kurtosis results were observed. Depending on the characteristics of the data, independent sample t-test, Mann-Whitney U test, or Pearson chi-square test were selected to evaluate the results. Kaplan-Meier survival analysis and log-rank test were used to evaluate long-term outcomes.

## Results

Age and sex

Of the 215 patients included in the study, 202 (93.95%) had ITN, and 13 (6.05%) had MSTN. Twenty-eight of the 286 procedures were performed on patients with MS (9.79%). The number of procedures performed in patients with ITN was 258 (90.21%). The mean age of patients with ITN was 58.18 (standard deviation {SD}: ±14.06), and the age range was 27-89. The mean age of patients with MSTN was 49.46 (SD: ±11.01), and the age range was 34-67. Of the 202 patients with ITN, 118 (58.42%) were females, and 84 (41.58%) were males. Of the 13 patients with MSTN, five (38.46%) were females, and eight (61.54%) were males (Table 2).

**Table 2:** Demographics of patients with trigeminal neuralgia (TN) treated with percutaneous radiofrequency thermocoagulation Values are presented as mean values±standard deviation (range) or number (%) F, female; M, male; R, right; L, left; B, bilateral; MSTN, trigeminal neuralgia secondary to multiple sclerosis

Characteristic	Value
Total patients (n=215)	
Age (years)	57.66±14.05 (27-89)
Sex, F/M	123 (57.21):92 (42.79)
Affected side, R/L/B	129 (60.00):79 (36.74):7 (3.26)
Idiopathic TN (n=202) (93.95)	
Age (years)	58.18±14.06 (27-89)
Sex, F/M	118 (58.42):84 (41.58)
Affected side, R/L/B	124 (61.39):75 (37.13):3 (1.48)
MSTN (n=13) (6.05)	
Age (years)	49.46±11.01 (34-67)
Sex, F/M	5 (38.46):8 (61.54)
Affected side, R/L/B	4 (30.77):5 (38.46):4 (30.77)

Painful side

**Figure 1:**
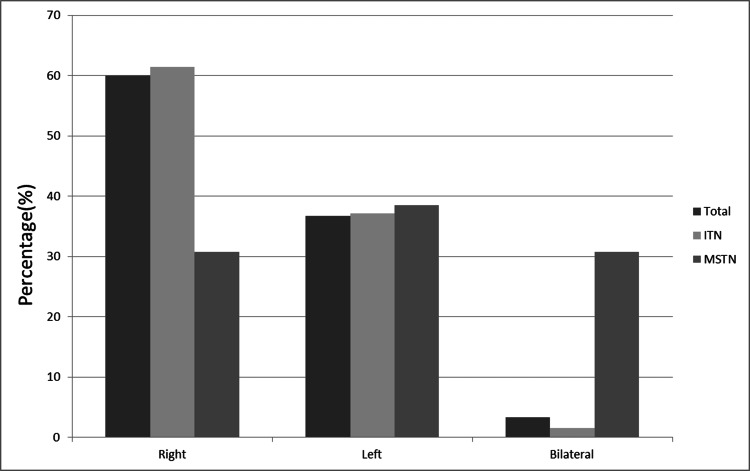
Painful side distribution in patients Comparisons are made in percentage (%) terms ITN, idiopathic trigeminal neuralgia; MSTN, trigeminal neuralgia secondary to multiple sclerosis

In patients with ITN, 124 (61.39%) had right-sided pain, 75 (37.13%) had left-sided pain, and three (1.48%) had bilateral pain. Among patients with MSTN, four (30.77%) had right-sided pain, five (38.46%) had left-sided pain, and four (30.77%) had bilateral pain (Table 2 and Figure 1).

Distribution of pain according to trigeminal nerve branches

**Figure 2:**
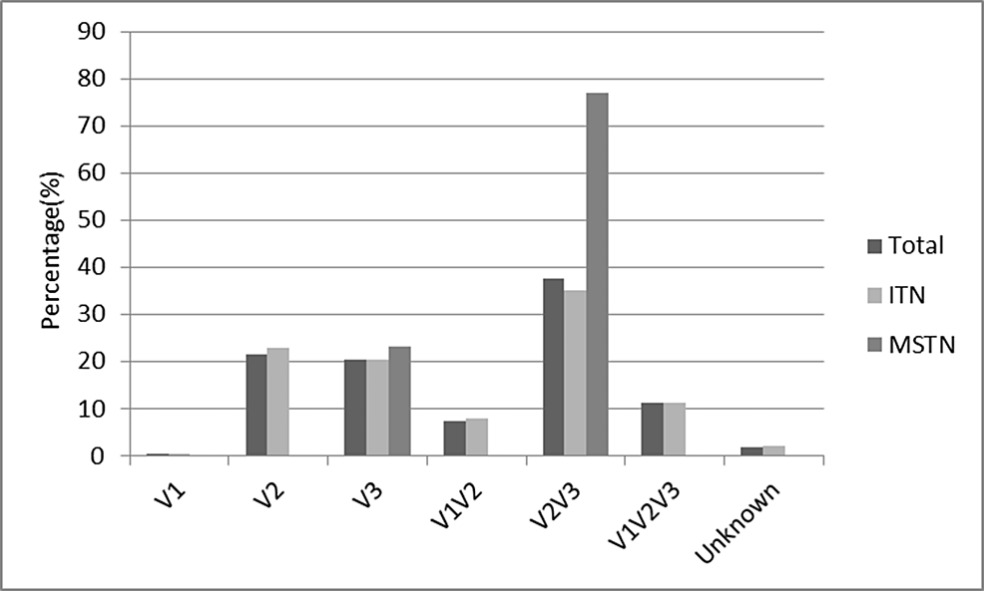
Localization of pain in patients treated with percutaneous radiofrequency thermocoagulation (215 patients) Comparisons are made in percentage (%) terms ITN, idiopathic trigeminal neuralgia; MSTN, trigeminal neuralgia secondary to multiple sclerosis

In patients with ITN, this distribution was V1 in one patient (0.49%), V2 in 46 patients (22.77%), V3 in 41 patients (20.30%), V1 and V2 in 16 patients (7.92%), V2 and V3 in 71 patients (35.15%), and V1, V2, and V3 in 23 patients (11.39%). The involved branch of four patients could not be identified (1.98%). In MSTN patients, this distribution was V3 in three patients (23.08%) and V2 and V3 in 10 patients (76.92%) (Table 3 and Figure 2).

**Table 3:** Localization of pain in patients treated with percutaneous radiofrequency thermocoagulation Values are the number of patients (%) ITN, idiopathic trigeminal neuralgia; MSTN, trigeminal neuralgia secondary to multiple sclerosis

Affected division	V1	V2	V3	V1 and V2	V2 and V3	V1, V2, and V3	The unknown	Total
All patients	1 (0.46)	46 (21.39)	44 (20.46)	16 (7.44)	81 (37.67)	24 (11.16)	4 (1.86)	215
ITN	1 (0.49)	46 (22.77)	41 (20.30)	16 (7.92)	71 (35.15)	23 (11.39)	4 (1.98)	202
MSTN	0	0	3 (23.08)	0	10 (76.92)	0	0	13

Past duration of symptoms

**Figure 3:**
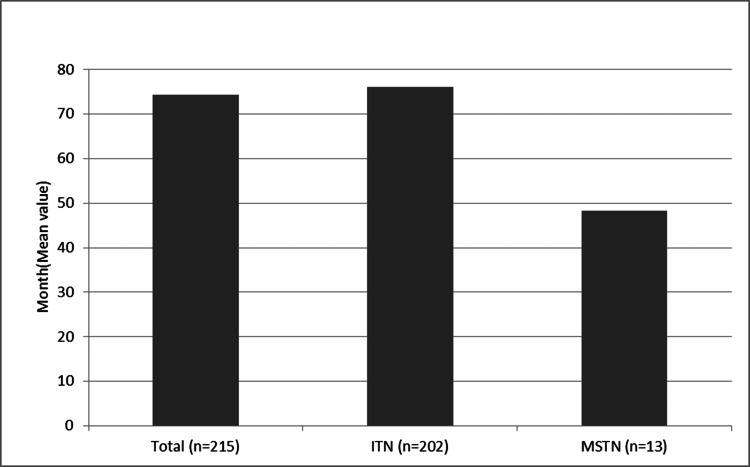
Past duration of the onset of complaints in patients Periods are calculated in months (mean values) ITN, idiopathic trigeminal neuralgia; MSTN, trigeminal neuralgia secondary to multiple sclerosis

The mean duration of symptoms in patients with ITN was 76.02 months (SD: ±75.71). The duration of complaints ranged from two to 480 months. The mean age at the onset of symptoms in patients with ITN was 51.46 (SD: ±14.51). The mean duration of symptoms in patients with MSTN was 48.23 months (SD: ±42.63) (Figure 3). The duration of complaints in this group ranged from five and 130 months. The mean age at the onset of symptoms in patients with MSTN was 46.31 (SD: ±8.58) (Table 4). The mean age at the onset of MS in these patients was 37.5 (SD: ±8.07). The mean interval between the diagnosis of MS and the onset of TN symptoms was 8.8 years (SD: ±4.71).

**Table 4:** Past duration and age of the onset of complaints in patients (mean values) Values are presented as mean value±standard deviation (range) ITN, idiopathic trigeminal neuralgia; MSTN, trigeminal neuralgia secondary to multiple sclerosis

	History of complaints (month)	Age of the onset of complaints
Total (n=215)	74.34 (74.43)	51.24 (14.24)
ITN (n=202)	76.02 (75.71)	51.46 (14.51)
MSTN (n=13)	48.23 (42.63)	46.31 (8.58)

Complications

**Figure 4:**
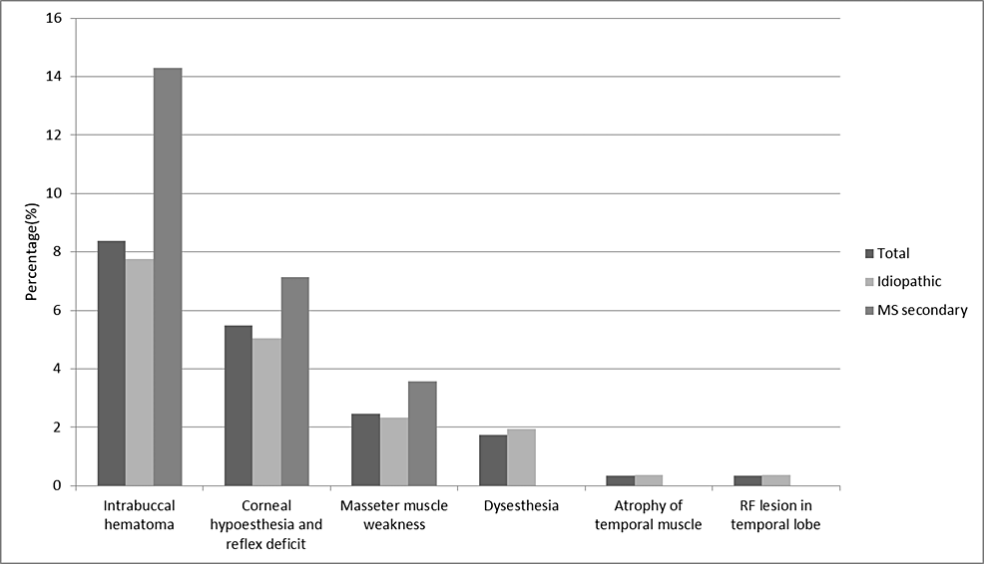
Complications after the 286 procedures performed Values are presented as percentage RF, radiofrequency; MS, multiple sclerosis

After 258 procedures in patients with ITN, intrabuccal hematoma was detected in 20 (7.75%) procedures, corneal hypoesthesia and reflex deficit in 13 (5.04%) procedures, masseter muscle weakness in six (2.33%) procedures, dysesthesia in five (1.94%) procedures, temporal muscle atrophy in one (0.39%) procedure, and 1 cm RF lesion in the temporal lobe in one (0.39%) procedure. Complications seen in patients with MSTN after 28 procedures were four (14.29%) intrabuccal hematoma, two (7.14%) corneal hypoesthesia and reflex deficit, and one (3.57%) masseter muscle weakness (Table 5 and Figure 4).

**Table 5:** Complications after the 286 procedures performed Values are the number of patients (%) ITN, idiopathic trigeminal neuralgia; MSTN, trigeminal neuralgia secondary to multiple sclerosis; RF, radiofrequency

Complication	Total (n=286)	ITN (n=258)	MSTN (n=28)
Intrabuccal hematoma	24 (8.39)	20 (7.75)	4 (14.29)
Corneal hypoesthesia and reflex deficit	16 (5.59)	13 (5.04)	2 (7.14)
Masseter muscle weakness	7 (2.45)	6 (2.33)	1 (3.57)
Dysesthesia	5 (1.75)	5 (1.94)	0
Atrophy of the temporal muscle	1 (0.35)	1 (0.39)	0
RF lesion in the temporal lobe	1 (0.35)	1 (0.39)	0

Early results after the procedures

**Figure 5:**
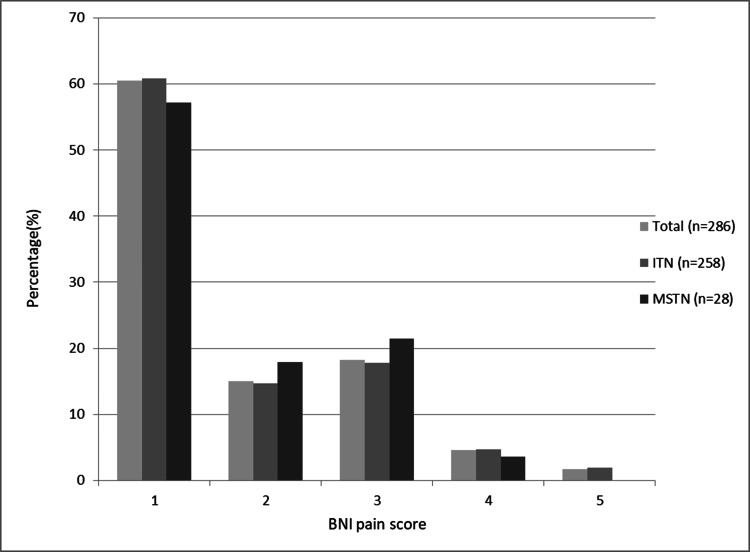
Early period results according to the Barrow Neurological Institute (BNI) pain intensity scale Comparisons are made in percentage (%) terms ITN, idiopathic trigeminal neuralgia; MSTN, trigeminal neuralgia secondary to multiple sclerosis

The outcomes in the early post-procedure period were evaluated according to the BNI pain scale. Of the 258 procedures performed in patients with ITN, 241 were in grades 1, 2, and 3 (93.41%). The number of results evaluated at grades 4 and 5 was 17 (6.59%). A total of 28 procedures were performed in 13 patients with MSTN. After these 28 procedures, the results of 27 patients were in grades 1, 2, and 3 (96.43%), while the treatment result of one patient was in grade 4 (3.57%) (Table 6 and Figure 5).

**Table 6:** Early RFT results according to the BNI scale (286 procedures) Values presented as number of procedures (%) RFT, radiofrequency thermocoagulation; BNI, Barrow Neurological Institute; ITN, idiopathic trigeminal neuralgia; MSTN, trigeminal neuralgia secondary to multiple sclerosis

BNI pain score	Total (n=286)	ITN (n=258)	MSTN (n=28)
1	173 (60.49)	157 (60.85)	16 (57.14)
2	43 (15.04)	38 (14.73)	5 (17.86)
3	52 (18.18)	46 (17.83)	6 (21.43)
4	13 (4.54)	12 (4.65)	1 (3.57)
5	5 (1.75)	5 (1.94)	0

Remission periods

**Figure 6:**
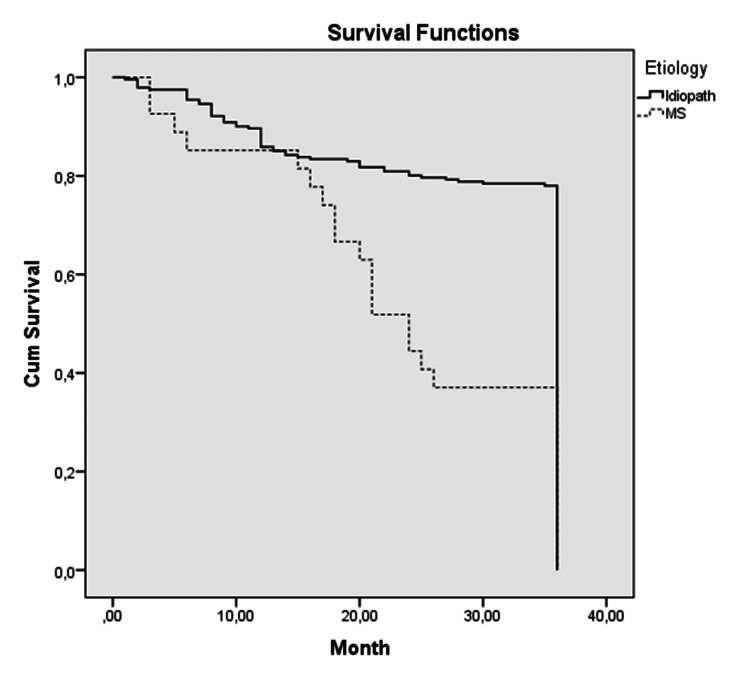
A graph showing the Kaplan-Meier analysis of the pain-free survival rate of the idiopathic group and the TN secondary to MS group treated by radiofrequency thermocoagulation The x-axis denotes pain-free survival in months, and the y-axis denotes cumulative survival Cum, cumulative; TN, trigeminal neuralgia; MS, multiple sclerosis

The remission periods of patients with ITN and MSTN after the procedures were evaluated over a period of 36 months. The evaluation included 268 procedures with BNI pain severity scores of 1, 2, and 3. Of these 268 procedures, 241 were in the ITN group, and 27 were in the MSTN group. After 36 months of follow-up, the mean remission time of the first group was 30.87 months, and the mean remission time of the second group was 23.81 months. Kaplan-Meier survival analysis was applied to the results (Figure 6).

## Discussion

Demographic characteristics of the patient groups

We compared the demographic characteristics of the patient groups. While the majority of females (58.42%) are in the ITN group, the majority of males (61.54%) are in the MSTN group. These results do not seem to be compatible with the literature. Females are expected to predominate as both MS and TN patients. In the study by Fallata et al. [13], 81.9% of patients with MSTN were females. Differently, in the study by Kanpolat et al., 64.7% of 17 MS patients with secondary TN were males, and 35.3% were females [14].

Age and pain history

In our series, the mean age of the first intervention was 58.18 for patients with ITN and 49.46 for patients with MSTN. It is seen that the mean age of patients diagnosed with MS is much lower. Whether the difference was significant or not was evaluated with the independent sample t-test method. As a result, the difference was found to be significant (p<0.0001). The reason for this was thought to be that MS mostly occurs in young adults [15,16].

The mean age of the onset of the first complaints of patients with ITN was 51.46, while the mean age of the onset of the first complaints of patients with MSTN was 46.31. Although there was a difference of 5.15 years between the mean age of the onset of the complaints of both groups, the statistical results were not significant (independent sample t-test, p=0.07).

The mean pre-procedure complaint history was 76.02 months (SD: ±75.71) in the ITN group and 48.23 months (SD: ± 42.63) in the MSTN group. When these results were subjected to Mann-Whitney U test, the difference was not found to be significant (p=0.182). What is remarkable in these results is the difference between the standard deviations (±75.71; ±42.63). These figures show that the range of distribution in the idiopathic group is wider than in the MS secondary group in terms of the occurrence of complaints. This is because the onset of TN complaints in the MS secondary group is closely related to the onset of MS [9].

The mean age of MS onset was 37.5 in patients with MSTN. When the literature was examined, it was stated that TN begins approximately 10 years after the first MS symptom in patients with MS. However, it was also reported in the literature that the age of the onset of TN may be the same or slightly younger than idiopathic TN, except when TN is the only MS symptom [14,17,18]. In the study by Fallata et al., the mean age of MS diagnosis of patients with TN neuralgia was 38.6 [13].

According to our results, the mean time between the diagnosis of MS and the onset of TN complaints was 8.8 years.

Localization of pain

The side of pain was compared between both groups. Accordingly, bilateral involvement was significantly higher in the MSTN group than in the ITN group. Percentages were 30.77%-1.48%. When the results were subjected to Pearson chi-square test, p<0.0001 was calculated. Previous studies have emphasized that bilateral TN is more common in patients with MSTN than in patients with ITN [3,7,14,17-20].

When the pain distribution is analyzed, it was observed that the involvement of V2-V3 branches is predominant in both groups. However, in the MS secondary group, it was observed that pain is significantly concentrated in V2-V3 branches. When we statistically investigated whether there was a relationship between pain distribution and etiological factor, we did not find a significant result (Pearson chi-square test, p=0.051).

In the series by Holland et al. [5], the majority involved the V2 branch. In the study by Jensen et al., V3 was predominant [20].

Complications

The complications after 286 procedures were intrabuccal hematoma, corneal hypoesthesia and reflex deficit, dysesthesia, masseter muscle weakness, temporal muscle atrophy, and RF lesion of 1 cm in the temporal lobe. There was no statistically significant difference between the complications seen in both groups (Pearson chi-square test, p=0.065). In addition, when we compared corneal hypoesthesia, reflex deficits, and temporal muscle atrophy, which we thought might be affected by MS, we did not find a significant difference between the two groups (Pearson chi-square test, p=0.63 and p=0.68).

Almost all patients who underwent the radiofrequency thermocoagulation procedure experienced hypoesthesia after successful interventions. This hypoesthesia was defined as "not disturbing and not troublesome" [21]. The uncomfortable sensory changes are described in the complication section under the definition of "dysesthesia." Donnet et al. declare that pain relief is obtained at the "cost" of trigeminal hypoesthesia, which ideally not only is purely thermalogic but also includes an almost inevitable tactile hypoesthesia or facial numbness [22].

Repetitive procedures

The percentage of multiple interventions in the same patient during the period included in the study was 20.29% in the idiopathic group and 61.54% in the group with MSTN. The difference between the groups was found to be statistically significant when subjected to Pearson chi-square test (p<0.0001). Similarly, in the article by Holland et al., it was reported that the rate of patients with MSTN who underwent second radiofrequency rhizotomy was 60% [5].

Results and follow-up

Among the total 286 interventions, 258 interventions were included in the ITN group. Twenty-eight procedures were performed in the MSTN group. According to the BNI pain scale, scores 1, 2, and 3 were considered successful. Accordingly, the rate of successful intervention in the idiopathic group was 93.10%, while this rate was 96.43% in the MSTN group. As can be seen, these results are very close to each other. When evaluated statistically, the results were not significant (Pearson chi-square test, p=0,90). When we look at the literature, the success rates in the MSTN group are 7/10 (70%) in the series by Holland et al. [5], 94.1% in the series by Kanpolat et al. [14], and 13/14 (92.86%) in the series by Hooge and Redekop [18].

Late results were evaluated over 36 months of follow-up. Thirty-six-month pain-free period was the maximum limit of follow-up. Every pain onset below 36 months was recorded. A total of 268 procedures with a BNI score of 1, 2, and 3 were evaluated. Of this number, 241 were in the idiopathic group and 27 in the MS group. The results of both groups were subjected to Kaplan-Meier survival analysis. According to these data, the pain-free period of the idiopathic group is significantly longer than the MS secondary group. When the results were evaluated according to log-rank test, the result proved to be significant (p<0.0001).

According to these results, the pain-free periods of patients with MSTN are significantly shorter than those of patients with ITN. Kanpolat et al. [14] reported complete pain relief rates as 92.7% in patients with ITN and 82.2% in patients with MSTN in their five-year follow-up study. In Brisman's article, it was reported that there was no significant difference between ITN and MSTN in terms of recurrent pain [17].

Limitations of the study

In our study, the number of patients in the MS secondary group was significantly low. However, the rate of the MS group in the patient population referred to our clinic is compatible with the rates given in the literature. Therefore, it was decided that this factor did not prevent the study.

In different studies, it has been reported that vascular compression is a factor that reveals in the clinic in the group with MSTN [7,23,24]. This will lead to a separate subgroup research in the MS secondary group. Our opinion is that this is the subject of another study in which radiological evaluations and treatment protocols are also discussed.

## Conclusions

In the results of this comparative retrospective cohort study, similarities and differences were found between both groups. The mean age of patients with MSTN was significantly lower than the mean age of the ITN group. The difference in clinical presentation was the predominance of bilateral pain type in the MS secondary group. There was no significant difference in the trigeminal nerve branch involved, and no consistent predominance has been reported in the literature. There was no difference between the two groups in terms of early treatment outcomes and complications. However, at long-term follow-up, the onset of post-procedure pain was significantly earlier in patients with MSTN. The RF thermocoagulation of the ganglion of Gasser in patients with typical TN is an effective, low-complication, and reproducible treatment modality. However, in the group of patients with MSTN, longer-acting treatment modalities should be investigated, taking into account the primary features that make the disease more sophisticated.
